# Special Issue: Microorganisms and Plant Nutrition

**DOI:** 10.3390/microorganisms9122571

**Published:** 2021-12-13

**Authors:** Nikolay Vassilev, Eligio Malusà

**Affiliations:** 1 Department of Chemical Engineering, University of Granada, C/Fuentenueva s/n, 18071 Granada, Spain; 2 Institute of Biotechnology, University of Granada, 18071 Granada, Spain; 3 The National Institute of Horticultural Research, 96-100 Skierniewice, Poland

Plant-beneficial microorganisms affect plant nutrition and health, as a key part of prebiotic-, probiotic-, and symbiotic-based interactions [1]. However, the application of soil microbial inoculants as biofertilizers and biopesticides in agriculture is still limited by factors related to their formulation, application method, and the lack of sufficient knowledge about the impact and interactions between microbial inoculants and native soil and plant host microbiomes [2,3].

Fertilization is crucial for sustainable agriculture as (bio)organic or chemical products change the soil microbiome, thus affecting plant growth, health, and productivity. A large study [4] showed that 21 years of fertilization significantly altered the prokaryotic and fungal communities in soil, whereas the influence of fertilization on the community of endophytes differed depending on the site (two geochemically and geographically distinct sites, with different environmental conditions and edaphic profile). At the site with cambisol, prokaryotic and fungal endophytes were significantly changed by addition of manure and sewage sludge while at the site with chernozem, neither the prokaryotic nor fungal endophytic communities were affected by fertilization treatments using the model plant *Solanum tuberosum* L. cv. Ditta. Manure, sewage sludge, and chemical fertilizer (NPK) significantly increased the relative abundance of the plant-beneficial bacteria *Stenotrophomonas*, *Sphingomonas* and the arbuscular mycorrhizal fungi. Fungal microorganisms, such as the P-solubilizing *Mortierella* and biocontrol *Basidiobolus* appeared with a higher relative abundance in sewage sludge treated soil. In general, fungal microorganisms are shown to simultaneously perform various important functions. *Aspergillus terreus* was reported to solubilize inorganic phosphates and promote plant growth and health but also to infect important crops [5] and therefore its form of application should be carefully assessed. However, the multifunctionality of *A. terreus* including its industrial importance shows the versatile nature of soil microorganisms.

Following the fungal theme in this Special Issue, we should particularly note mycorrhizal symbioses, which represent a very efficient tool for improving plant nutrient uptake and productivity, allowing a reduction in fertilizer inputs [6]. Plant mycorrhization measured in agri-soil is related to the mycorrhizal functional trait of weeds. It was shown that applying agroecological service crops in cropping systems determines changes in the weed community. This affects the development of the mycorrhizal mycelial network in the rhizosphere, which favors the crop of interest. Cereals, used as green mulches or intercropped, may drive the weed selection in favor of species supporting mycorrhiza, and promote the mycelial network. This significantly increases mycorrhization, the P uptake, the yield and quality traits of the cash crop. Accordingly, bacterial endophytic strains promote plant growth, increasing shoot and root biomass, plant nutritional status, and the use efficiency of most nutrients. In a study with sugarcane plant models, inoculation with six endophytic plant growth promoting bacteria (PGPB) induced changes at the biochemical level, and provoked changes in the foliar free amino acid and polyamine relative concentrations of citrulline, putrescine, glycine, alanine, glutamate, glutamine, proline, and aspartate [7]. The results suggested that PGPB-inoculation establishes a symbiotic association between sugarcane seedlings and endophytic bacteria, which also results in lower plant stress compared to non-inoculated plants. The general conclusion of this study is that the ability of the endophytic strains to promote sugarcane growth could be attributed to different mechanisms that modulate N metabolism and nutrient use efficiency. A mechanism that affects plant N nutrition was described in the invasive plants *Fallopia* spp., which exude secondary metabolites called procyanidins that inhibit microbial denitrification activity [8]. This phenomenon, called biological denitrification inhibition (BDI), allows plants to limit the nitrate consumption of denitrifiers and hence, supports plants in using the nitrate for their own nutrition and growth. Procyanidins are tannic molecules highly represented in the plant world, derived from the metabolic pathway of anthocyanins—compounds omnipresent in the secondary metabolism of plants. This work demonstrated that the addition of procyanidins inhibits denitrifiers, which caused increases in the soil nitrate level, inducing an improvement in morphological traits of the model plant (celery, *Apium graveolens* L. var TANGO, Bejo). Procyanidin amendment provoked the lowest nitrogen concentration in plant tissues and reduced N_2_O emissions.

Modulation of plant growth and metabolism and improvement of the fertility status of the rhizosphere soils was reported in a work studying the effect of plant growth promoting rhizobacteria (PGPR) in the revegetation and rehabilitation of rainfed areas by [9]. The application of PGPR and salicylic acid (SA) individually or as a combined treatment significantly enhanced the indole-3-acetic-acid (73%) and gibberellic acid (70%) contents but decreased (55%) the abscisic acid content of shoots under natural conditions. The combined treatment of the PGPR and SA alleviated the adverse effects of low moisture stress in rainfed soil areas and increased accumulation of leaf chlorophyll content, chlorophyll fluorescence, and carotenoids in the shoots of maize used as a model. Significant increases in the organic matter content were noted, as well as in the contents of Ca, Mg, K Cu, Co, Fe and Zn in the shoots of plants and rhizosphere of maize inoculated with the PGPR consortium. 

The effect of environmental conditions, including soil, biotic and abiotic factors, whether in combination with the specific influence of the plant species is of great importance in the soil microbial community profile. There is an urgent need to determine/distinguish the effect of plant vs. edaphic factors. Similarly, a different approach is needed to separate the individual effect of the plant community other than correlational research, which is usually adopted for environmental factors. Analyzing responses of unique bacterial operational taxonomic units (OTUs) to specific plants is useful to understand the individual effect of the plant community. Our colleagues from Korea tried to respond to the above challenges and, in general, to determine how bacterial community characteristics and functions are influenced by the plant community and how they are associated with soil chemical properties [10]. In their study, bacterial responses were compared in five plant communities (two forage and three weed, where >65% of the coverage was by one or two species) having different dominant species. 16S rRNA sequencing was used to identify bacteria and to infer, in silico, their metabolic features. Uniquely responsive bacterial OTUs were analyzed to discriminate the effect of plant community on bacteria from that of soil chemical properties. This work describes how changes in species dominance in the plant community drive spatial variation in soil bacterial community characteristics and functions in association with edaphic conditions. Separately analyzing the effects of both plant communities and soil chemical properties on the bacterial community is crucial for understanding the biogeographic process at a small scale.

This Special Issue also includes a comparison between different fermentation processes used to produce plant-beneficial microorganisms [11]. Liquid submerged fermentation and solid-state fermentation can be used depending on the microbial characteristics, the formulation scheme, and the mode of application. Total biomass, spore production, and cell-free liquid are the main final products, independently of the mode of fermentation, but each one of these products needs optimal conditions to maintain its viability and efficacy during storage and in the field. The Special Issue presents a small portion of the work on microbe–plant interactions, but it reflects the dynamic and great progress of methods and approaches applied in this field.
